# Stapedial reflex threshold predicts individual loudness tolerance for people with autistic spectrum disorders

**DOI:** 10.1007/s00221-018-5400-6

**Published:** 2018-10-11

**Authors:** Yoshiyuki Ohmura, Itsuki Ichikawa, Shinichiro Kumagaya, Yasuo Kuniyoshi

**Affiliations:** 1 Department of Mechano-Informatics, Graduate School of Information Science and Technology, The University of Tokyo, 7-3-1 Hongo, Bunkyo-ku, Tokyo, 113-8656 Japan; 2 Research Center for Advanced Science and Technology, The University of Tokyo, 4-6-1, Komaba, Meguro-ku, Tokyo, 153-8904 Japan

**Keywords:** Autism, Hyperacusis, Stapedial reflex, DPOAE

## Abstract

People with autism spectrum disorder (ASD) frequently show the symptoms of oversensitivity to sound (hyperacusis). Although the previous studies have investigated methods for quantifying hyperacusis in ASD, appropriate physiological signs for quantifying hyperacusis in ASD remain poorly understood. Here, we investigated the relationship of loudness tolerance with the threshold of the stapedial reflex and with contralateral suppression of the distortion product otoacoustic emissions, which has been suggested to be related to hyperacusis in people without ASD. We tested an ASD group and a neurotypical group. The results revealed that only the stapedial reflex threshold was significantly correlated with loudness tolerance in both groups. In addition to reduced loudness tolerance, people with lower stapedial reflex thresholds also exhibited higher scores on the Social Responsiveness Scale-2.

## Introduction

Hyperacusis is a disorder characterized by excessive responses to auditory stimulation. The stapedial reflex (SR) has been extensively studied as a means of quantifying the degree of hyperacusis (Olsen 1999). Although the threshold of this reflex has been reported to be lower in people with a relatively weak tolerance for loud noise (McCandless and Miller 1972; Greenberg and Mcloed 1979; Olsen 1999; Al-Azazi and Othman 2000), several previous studies have not supported this relationship (Denenberg and Altshuler 1976; Holmes and Woodford 1977; Ritter et al. 1979). This discrepancy can be explained by inconsistent factors in experimental design, such as the stimuli used or the hearing function of the participants (Al-Azazi and Othman 2000). More recently, Knudson et al. (2014) observed that contralateral suppression of distortion product otoacoustic emissions (DPOAE) is increased in patients with hyperacusis. While the mechanisms underlying this excessive DPOAE suppression are unknown, these results suggest that inner ear function can predict hyperacusis.

A number of studies have reported abnormal responses to auditory stimuli among people with autistic spectrum disorder (ASD). Although ASD patients commonly exhibit both hypersensitivity to auditory stimuli and cases of hypoactivity, it is primarily hypersensitivity that it is considered a problem in daily life (Elwin et al. 2012; Robertson and Simmons 2015). More than half a century ago, Kanner noted that people with autism showed excessive reactions to specific sounds (Kanner 1943). More recently, Khalfa et al. (2004) reported that, compared with typically developing (TD) participants, people with ASD exhibited stronger discomfort in response to auditory stimulation. However, it remains unclear whether SR threshold or DPOAE can be used as markers for the degree of auditory sensitivity in people with ASD.

Although several studies have reported that SR threshold in children with ASD does not differ significantly from that in TD children (Gomes et al. 2004; Gravel et al. 2006; Tharpe et al. 2006), a more recent study found significant differences in the low-frequency band (Lukose et al. 2013). In addition, the reported differences in DPOAE suppression between ASD and TD are also controversial, because they are not always observed, even under similar experimental conditions (Danesh and Kaf 2012; Kaf and Danesh 2013). Furthermore, the relationship between hyperacusis in ASD and inner ear function remains poorly understood.

Tolerance levels for pure-tone sound have frequently been used to evaluate hyperacusis. However, in the current study, we used speech sounds as stimuli, because sensitivity for speech sound is higher than that for pure tones (Smith and Bennetto 2007). Furthermore, we used a visual analog scale to evaluate hyperacusis using a finer scale, rather than a binary measure.

To the best of our knowledge, no previous study has examined the relationship between loudness tolerance and either SR threshold or DPOAE suppression in ASD. In the current study, we examined these relationships to establish a new marker for hyperacusis in ASD.

## Methods

### Participants

21 healthy adults (TD group) and 14 people with ASD (ASD group) participated in the current study. Members of the TD group were recruited by a temporary employment agency. Members of the ASD group were recruited online and had ASD or another relevant disorder [pervasive developmental disorders (PDD), attention deficit (AD), high functioning autism (HFA), Asperger’s syndrome, and pervasive developmental disorders—not otherwise specified (PDD-NOS)]. The two groups were matched for age and sex (Table 1). There were no eligibility criteria. Subjects have self-reported on the diagnosis of ASD or other relevant disorders, and one of the authors confirmed the medical certificate.

**Table 1:** Participant demographics

Measure	ASD group	TD group
Group size	14	21
Male	7	10
Female	7	11
Average age	41.3 ± 6.3 (30–53)	42.0 ± 7.0 (30–54)
Social Responsiveness Scale-2 (SRS-2)	118 ± 26.6 (62–158)	52.9 ± 15.5 (21–82)

To assess autistic traits, each participant completed the Social Responsiveness Scale-2 (SRS-2) (Constantino and Gruber 2012). A significant group difference in SRS-2 score was found using Welch’s *t* test (*t*_(19)_ = 8.26, *p* < 0.001).

### Apparatus

All experimental procedures were conducted in a soundproof room (YAMAHA, Shizuoka, Japan). The amount of noise attenuation in the soundproof room was 15 dB, as measured by an NL-27K-1822 noise meter (Rion, Tokyo, Japan).

SR threshold and DPOAE were measured using a tympanometer (Titan, DiaTec Company, Kawasaki Saiwai-ku, Kanagawa, Japan). Before the experiment, we selected an earpiece (Titan, Diatec) for each participant to prevent air leaks. Aside from the stimuli used to induce the SR and DPOAE, all other auditory stimuli were delivered via an audio interface (DUO-CAPTURE-EX, Roland Corporation, Hamamatsu Kita-ku, Shizuoka, Japan) using ear phones (ATH-CKS55XBK, Audio-Technica Corporation, Machida, Tokyo, Japan). We controlled the Titan devices using software (Otoaccess, Diatec) run on a Windows 8 platform. We administered the auditory stimuli using custom software developed in Scilab v5.5.2 (ESI company, Avenue de Suffren, Paris, France) for Windows 8.

### Experimental procedure

We measured the SR threshold and DPOAE for all participants and obtained subjective loudness evaluations. All measurements were performed in the left ear first and then in the right ear. Each left and right ear measurement was first made, while no sound was applied to the contralateral ear. Afterward, both measurements were repeated with broad band noise ranging from 0.4 to 5 kHz applied to contralateral ear. For the SR threshold, we used the average of the data measured under both conditions, with and without contralateral noise.

**Fig. 1:**
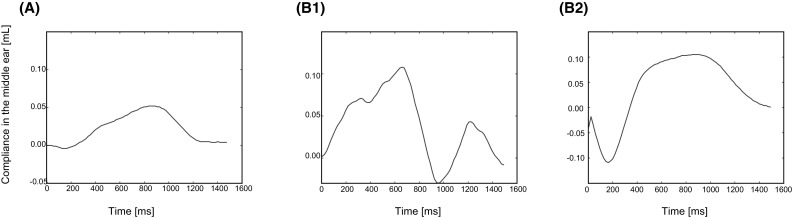
Examples of SR waveforms. Waveforms similar to that shown in graph A were regarded as reliable, while waveforms similar to those shown in graph B1 or B2 were regarded as unreliable

To keep the duration of the experiment within 1 h, we did not repeat measurements even if the results were not reliable. To compensate for this, we excluded any unreliable data from the analysis. Regarding SR, the waveforms obtained from the measurement were regarded as unreliable if they were noisy or non-smooth (Fig. 1). For DPOAE, we used data when the reliability values calculated by Titan software were higher than 98%.

### Stapedial reflex threshold

SRs were induced by 0.5, 1, 2, 3, and 4 kHz pure tones. The inducing stimulus was first presented at a gain of 65 dB, which was incremented in steps of 3 dB until the SR was detected or until the stimulus reached the maximum level that could be induced by the apparatus.

We removed unreliable SR waveforms when the time-series of acoustic impedance was either noisy or non-smooth (Fig. 1). Furthermore, if SR thresholds could not be detected using a stimulus at the maximum dB level, we set the SR threshold to be the maximum level. The proportion of missing values of SR threshold was 22.0% of the total number of measurements.

### DPOAE

We recorded the power level (DPLV), signal-to-noise ratio (SNR), and noise level of the DPOAE. The sound pressure levels (SPL) for the primary and secondary tones (*L*1 and *L*2) were fixed, so that *L*2 = *L*1–10 dB. The frequencies of the two tones (*f*1 and *f*2) were fixed, so that *f*2/*f*1 = 1.22 Hz. Measurement conditions are summarized in Table 2. The proportion of missing values of DPOAE was 37.19% of the total number of measurements.

**Table 2:** DPOAE measurement conditions

Frequency (Hz)		Pressure level (dB SPL)	
*f*2	*f*1	*L*1	*L*2
500	410	65	55
1000	820		
1500	1230		
2000	1639		
3000	2459		
4000	3279		
5000	4098		
6000	4918		
7000	5738		
8000	6557		
1068	875	40	30
		45	35
		50	40
		55	45
		60	50
		65	55
		70	60
		75	65
		80	70
3098	2539	40	30
		45	35
		50	40
		55	45
		60	50
		65	55
		70	60
		75	65
		80	70

### Subjective loudness evaluation

Participants heard several speech stimuli and were asked to evaluate the loudness using a visual analog scale from 0 (silent) to 10 (unbearably loud). We used four voices from the Online gaming voice chat corpus with emotional label (OGVC) provided by the Speech Resources Consortium at the National Institute of Informatics (NII-SRC, Hitotsubashi, Chiyoda-ku, Tokyo, Japan). Based on a previous report that speech stimuli elicit a lower response sound pressure than pure-tone stimuli in children with autism (Smith and Bennetto 2007), we predicted that speech stimuli would be better suited for examining auditory hypersensitivity. Since all participants in this experiment were Japanese, we used a Japanese speech corpus. OGVC was selected, because it does not include dialects, and included variations such as emotion representation.

We selected two surprise voices, one angry voice, and one fearful voice, which were spoken by actors, from the OGVC. We prepared the stimuli for each voice at five decibel levels (0, 5, 10, 15, and 20 dB).

Each participant completed four sessions. Each session included five randomly chosen stimuli that were presented from low to high volume. If participants could not stand the loud sounds, they were able to decrease the number of stimuli per session. As a result, two participants from the ASD group skipped stimuli ≥ 15 dB and 17 participants from both groups skipped stimuli ≥ 20 dB. Therefore, missing data constituted 6.36% of the total number of measurements.

### Data analysis

#### DPOAE suppression

We analyzed DPLV when the Titan tympanometer indicated that reliability was greater than 98%. The change in DPLV elicited by contralateral noise was quantified as a magnitude ratio (no noise/noise) and expressed in decibels. The mean change for each condition was calculated for each ear. We analyzed the magnitude change for each condition when a significant suppression (or facilitation) of DPLV was identified. Mean magnitude changes were considered significant when their 95% confidence intervals excluded zero (Knudson et al. 2014).

#### Normalized mean

Because of missing values, the amount of usable data was not always the same across conditions. To compensate for this and to allow us to explain overall trends among the participants, we calculated the normalized mean for each parameter (SR thresholds, DPOAE suppression, and loudness-evaluation scores). First, we normalized all the data, so that the mean was 0 and the standard deviation was 1 for each measurement condition. We then averaged all data from each ear of each participant. The value resulting from this procedure was defined as the normalized mean.

#### Hyperacusis index

We defined the hyperacusis index for each participant as the normalized mean of the subjective loudness-evaluation score from the subjective loudness-evaluation task.

#### Statistical analysis

For each variable, we conducted a two-factor mixed-design analysis of variance (ANOVA), with Group (TD vs. ASD) and Ear (left vs. right) as the factors. Post hoc comparisons were conducted using the Bonferroni method. Correlations were analyzed using Spearman’s correlation coefficients. Statistical significance was set at *p* < 0.05.

## Results

### SR threshold can be an indicator of hyperacusis regardless of group

**Fig. 2:**
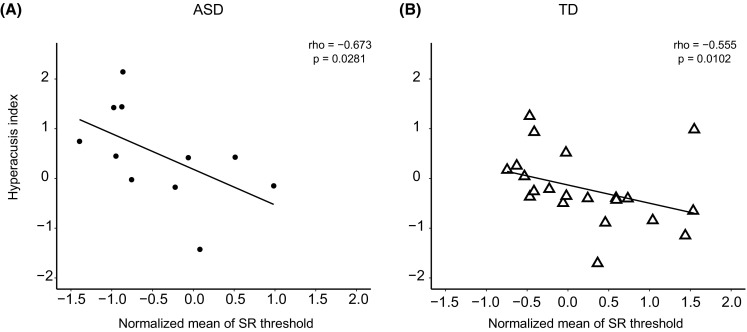
Scatter plot showing the normalized mean-SR threshold vs. the hyperacusis index. Note the negative correlation for both the **a** ASD and **b** TD groups

**Fig. 3:**
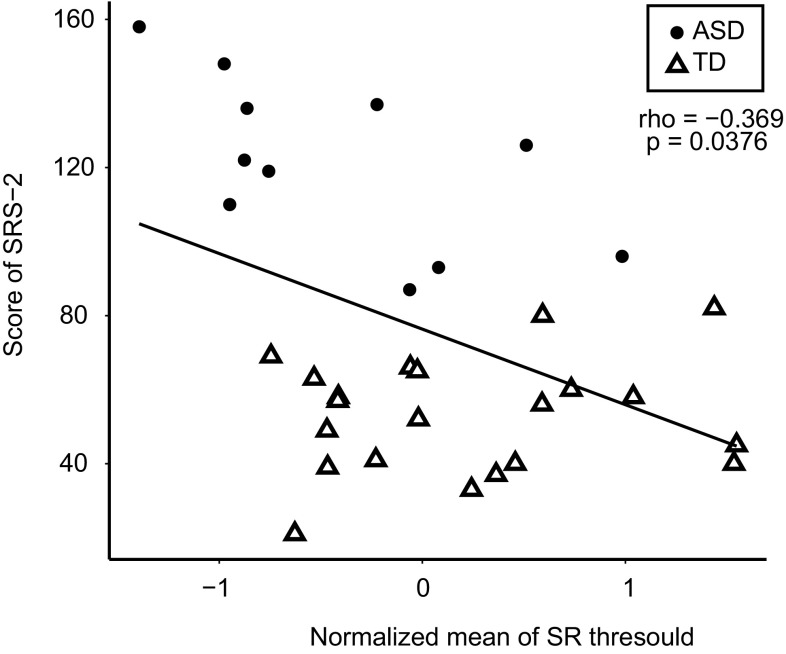
Correlation between SRS scores and the normalized mean-SR threshold

For both groups, we examined whether the SR threshold or DPOAE suppression was correlated with the hyperacusis index. We removed unreliable SR thresholds from the analysis (see “Methods”). The number of samples and the correlation results for the SR threshold are summarized in Table 3. Correlation coefficients in all the conditions were negative, but statistical significance was limited to: 1 kHz in the ASD group (both ears), 1 kHz in the TD group (left ear), 2 kHz in both groups (right ear), and 3 kHz in the TD group (left ear). For both groups, the normalized mean-SR thresholds were significantly correlated with the hyperacusis index (ASD group: *ρ* = − 0.673, *p* = 0.0281; TD group: *ρ* = − 0.555. *p* = 0.0102. Fig. 2), indicating that, while SR threshold under specific conditions is not a good indicator of hyperacusis, the normalized mean threshold across multiple conditions was a predictive index for hyperacusis. In addition, we observed a significant negative correlation between SRS-2 scores and the normalized mean-SR threshold across conditions (*ρ* = − 0.369, *p* = 0.0376, Fig. 3). We also observed an almost significant correlation between SRS-2 scores and the normalized mean-SR threshold in the ASD group (*ρ* = − 0.609, *p* = 0.052), but not in the TD group (*ρ* = 0.0630, *p* = 0.786).

**Table 3:** Numbers of samples used in each SR threshold-measurement condition and the corresponding correlations between the SR threshold and the hyperacusis index

Frequency (Hz)	Number of used data	Ear	Group	Correlation withhyperacusis index	
				*p* value	*ρ*
500	15	Left	ASD	0.329	− 0.398
	36		TD	0.0773	− 0.404
	19	Right	ASD	0.279	− 0.359
	37		TD	0.174	− 0.308
1000	18	Left	ASD	0.0392*	− 0.656
	36		TD	0.0222*	− 0.508
	18	Right	ASD	0.00266***	− 0.807
	38		TD	0.382	− 0.207
2000	19	Left	ASD	0.137	− 0.478
	35		TD	0.0419*	− 0.459
	20	Right	ASD	0.0299*	− 0.651
	38		TD	0.101	− 0.368
3000	18	Left	ASD	0.136	− 0.479
	35		TD	0.00600**	− 0.592
	20	Right	ASD	0.569	− 0.193
	34		TD	0.0633	− 0.434
4000	17	Left	ASD	0.125	− 0.518
	35		TD	0.0979	− 0.380
	20	Right	ASD	0.0838	− 0.544
	38		TD	0.254	− 0.260

*p < 0.05; **p < 0.01; ***p < 0.005

For DPOAE suppression, the conditions resulting in a significant suppression (or facilitation) were limited (Table 4). Analysis of these conditions indicated that DPOAE suppression did not correlate significantly with the hyperacusis index (Table 5). Furthermore, similar results were obtained for the normalized mean-DPOAE suppression across conditions (ASD group: *ρ* = 0.112, *p* = 0.733; TD group: *ρ* = − 0.181, *p* = 0.458), indicating that the normalized mean-DPOAE suppression was not a good predictor of hyperacusis.

**Table 4:** Number of samples of each measurement condition for DPOAE contralateral suppression

Frequency (Hz)		Pressure level (dB SPL)		Number of reliable data		Significancy of contralateral suppression
*f*2	*f*1	*L*1	*L*2	ASD group	TD group	
*Left ear*						
500	410	65	55	4	6	
1000	820			10	16	
1500	1230			11	18	
2000	1639			11	18	
3000	2459			10	18	
4000	3279			11	18	
5000	4098			11	20	
6000	4918			8	17	
7000	5738			8	17	
8000	6557			7	13	
1068	875	40	30	0	2	
		45	35	0	5	
		50	40	2	10	*
		55	45	2	16	
		60	50	11	17	
		65	55	11	17	
		70	60	11	18	
		75	65	11	18	**
		80	70	12	18	
3098	2539	40	30	0	6	
		45	35	2	10	*
		50	40	2	14	
		55	45	2	16	*
		60	50	11	17	
		65	55	12	19	*
		70	60	12	19	
		75	65	12	19	
		80	70	12	20	
*Right ear*						
500	410	65	55	6	9	
1000	820			11	17	
1500	1230			10	17	
2000	1639			11	17	
3000	2459			12	17	
4000	3279			11	18	
5000	4098			12	18	
6000	4918			10	17	
7000	5738			11	17	
8000	6557			9	17	
1068	875	40	30	0	5	
		45	35	0	7	
		50	40	1	13	*
		55	45	1	15	
		60	50	10	16	*
		65	55	10	15	
		70	60	10	17	*
		75	65	9	17	**
		80	70	10	18	*
3098	2539	40	30	1	8	
		45	35	1	12	
		50	40	1	14	
		55	45	3	16	
		60	50	12	17	
		65	55	12	17	
		70	60	12	19	
		75	65	12	19	
		80	70	11	19	

*p < 0.05; **p < 0.01

**Table 5:** Measurement ranges in which a significant DPOAE contralateral suppression was detected

*f*2 (Hz)	*L*1 (dB SPL)	Group	Correlation with hyperacusis index	
			*p* value	*ρ*
*Left ear*				
1068	50	ASD	NA	NA
		TD	1.00	0.00606
	75	ASD	0.595	0.182
		TD	0.375	− 0.222
3098	45	ASD	NA	NA
		TD	0.126	0.517
	55	ASD	NA	NA
		TD	0.568	0.155
	65	ASD	0.640	0.151
		TD	0.606	0.126
*Right ear*				
1068	50	ASD	NA	NA
		TD	0.683	− 0.126
	60	ASD	0.865	− 0.0667
		TD	0.816	− 0.633
	70	ASD	0.707	− 0.139
		TD	0.775	0.0748
	75	ASD	0.966	− 0.0167
		TD	0.940	− 0.0196
	80	ASD	0.113	0.539
		TD	0.754	− 0.0796

*p < 0.05

### Both hyperacusis and lower SR threshold are exhibited by the ASD group

**Fig. 4:**
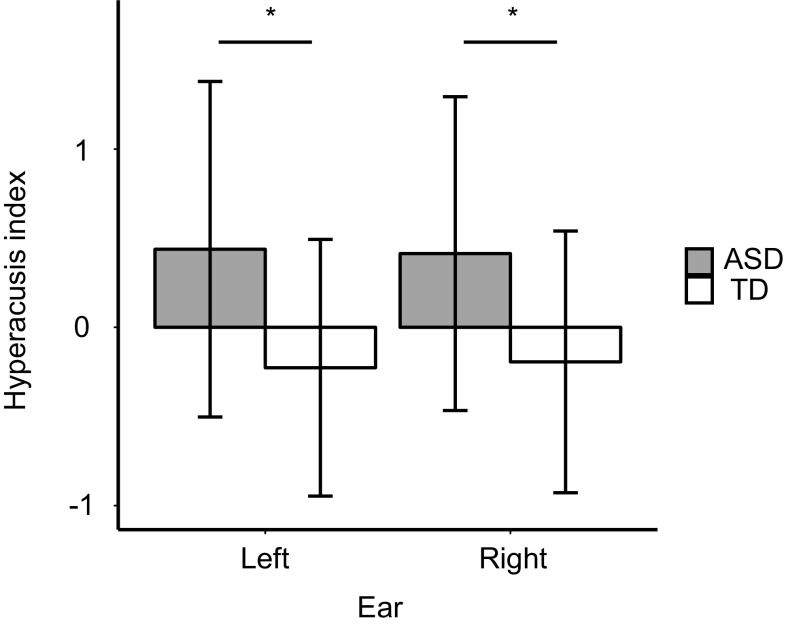
Comparison of normalized mean subjective loudness between groups for each ear. Values shown are mean ± SE (*p < 0.05)

The two-factor ANOVA revealed a main effect of group on the hyperacusis index (ASD: mean = 0.426; TD: mean = − 0.21; *F*_(1,33)_ = 5.50, *p* = 0.0251; Fig. 4), indicating that hyperacusis was significantly greater in the ASD group. We did not find a main effect of Ear (*F*_(1,33)_ = 0.0049, *p* = 0.945) or any interaction (Group × Ear: *F*_(1,33)_ = 0.231, *p* = 0.634). These results indicate that the ASD group had less tolerance for loud noises than the TD group.

**Fig. 5:**
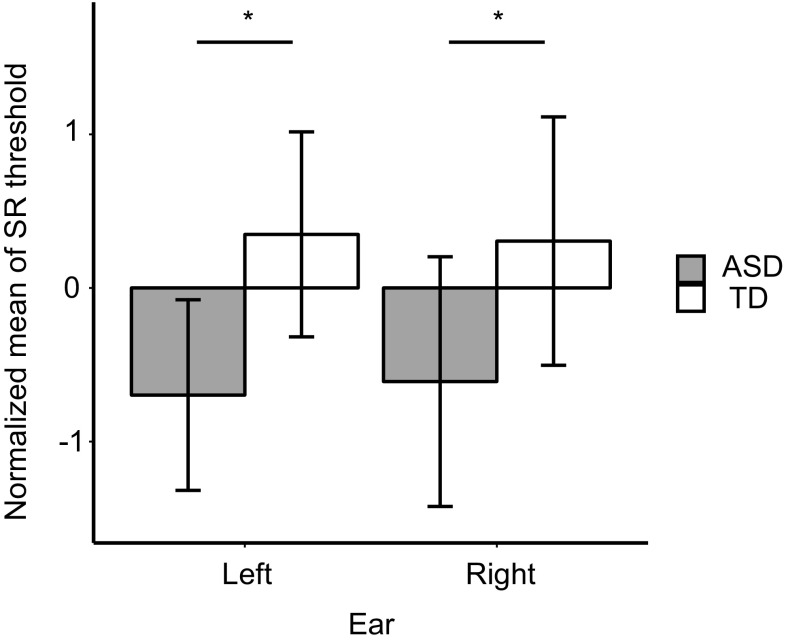
Comparison of the normalized mean-SR threshold between the two groups for each ear. Values shown are mean ± SE (*p < 0.05)

**Fig. 6:**
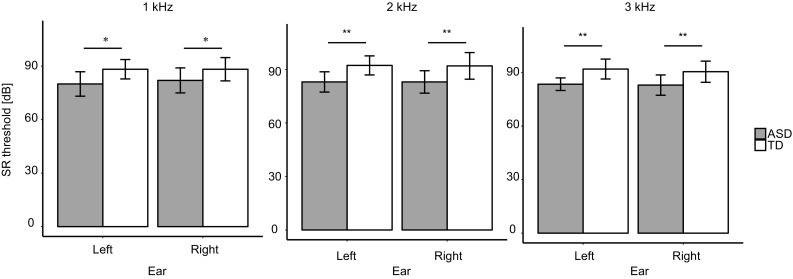
Comparison of the SR threshold for 1 kHz (**a**), 2 kHz (**b**), and 3 kHz (**c**) stimuli. Values shown are mean ± SE (**p < 0.01, *p < 0.05)

Consistent with these results, we also found a main effect of group on the normalized mean-SR threshold across conditions (ASD: mean = − 0.654; TD: mean = 0.327; *F*_(1,16)_ = 7.68, *p* = 0.0136; Fig. 5), indicating that SR threshold was significantly lower in the ASD group. Analysis revealed no main effect of ear (*F*_(1,16)_ = 0.0478, *p* = 0.8298) and no significant interaction (*F*_(1,16)_ = 0.430, *p* = 0.5214). Furthermore, SR threshold was significantly lower in the ASD group in the 1–3 kHz range (1 kHz: ASD = 81.0, TD = 88.3, *F*_(1,16)_ = 6.38, *p* = 0.0225; 2 kHz: ASD mean = 83.0, TD mean = 92.1, *F*_(1,16)_ = 10.9, *p* = 0.0045; 3 kHz: ASD mean = 83.3, TD mean = 91.3, *F*_(1,16)_ = 10.7, *p* = 0.0048, Fig. 6).

These results suggest that hyperacusis was a more prominent feature in ASD than in TD individuals, and that SR threshold was an appropriate index for examining hyperacusis in ASD.

## Discussion

In the current study, we examined whether inner ear function could explain hyperacusis in ASD. First, we determined how well the hyperacusis index correlated with the SR threshold and with DPOAE suppression, in both the ASD and TD groups. We used this method, because hearing ability affects the relationship between the hyperacusis index and these measurements. We found that only SR threshold was correlated with the hyperacusis index in both groups. In addition, we found that the SR threshold for the ASD groups was lower than that for the TD group. Finally, we found that the SR threshold was correlated with sociality index scores. Taken together, these results suggest that hyperacusis in ASD can be explained by a highly sensitive inner ear.

We examined the SR threshold in adults, and observed that it was lower in people with ASD compared with TD individuals. In a previous study, children with ASD were also reported to have lower SR thresholds (Lukose et al. 2013). However, some previous studies reported no significant difference in SR threshold between children with and without ASD (Tharpe et al. 2006; Gravel et al. 2006). Differences in the age range of the included subjects may have caused the inconsistent results between the previous studies. In addition, several previous studies suggested that SR thresholds are affected by aging (Silman 1979; Gelfand and Piper 1981; Silverman et al. 1983) and early development (Mazlan et al. 2007). The results of the present study indicate that a low SR threshold can provide an indicator for hyperacusis among young adults. However, it is necessary to conduct experiments with the other age groups to confirm the generalizability of the SR threshold as an index of hyperacusis.

In contrast, the current results did not confirm a relationship between the hyperacusis index and DPOAE contralateral suppression in either group. These findings are inconsistent with the results of Knudson et al. (2014), but can be explained by differences in pathology and measurement conditions. DPOAE contralateral suppression occurs most prominently at the peak point of the DPOAE fine structure (Reuter and Hammershed 2006), suggesting that the relationship between suppression and frequency involves negligible inter-individual differences. Thus, because of such variability, DPOAE contralateral suppression may not provide a suitable method for evaluating hyperacusis. However, to detect the peak of the DPOAE fine structure, a frequency resolution of 10–20 Hz is required (Sun 2008). Therefore, it is possible that DPOAE contralateral suppression could not be evaluated properly using our measurement method.

The SR is affected not only by the function of the inner ear but also by the brainstem auditory circuit (e.g., the cochlear nuclei and the superior olivary complex) (Lukose et al. 2013, 2015; Kulesza and Mangunay 2008; Kulesza Jr et al. 2011), suggesting that people with ASD may exhibit abnormalities in the brainstem (Klin 1993; Hashimoto et al. 1995). However, we cannot rule out the possibility that the cortical projections involve the SR threshold, because auditory activity can be modulated by the auditory cortex (Khalfa et al. 2001).

The current study involved several potential limitations that should be considered. First, we tested a relatively small sample size. Because ASD is a heterogeneous neurological disorder, the generalizability of the current results should be confirmed in a larger ASD population. However, we observed a negative correlation between hyperacusis and SR threshold in both the TD group and the ASD group, indicating that this relationship is not sensitive to the heterogeneity in ASD. Second, we did not counterbalance the left and right ears in the current experiments, because laterality was not our focus. Thus, in the current study, we were unable to observe any laterality, and further examination is required to elucidate this issue. Third, as mentioned above, we cannot rule out the possibility that our DPOAE measurement was not sufficient for evaluating hyperacusis, because DPOAE contralateral suppression must be measured at the peak point of DPOAE fine structure. To achieve this, DPOAE must be measured repeatedly with a high-frequency resolution. Thus, we propose that the SR threshold provides a more practical way to evaluate hyperacusis than the DPOAE.

Finally, the current results confirmed that people with ASD have a reduced tolerance for loudness compared with TD individuals, and that the SR threshold was significantly correlated with sociality scores. Because the SR threshold can be easily measured even in small children, it could provide a suitable marker for detecting ASD at the early stages. To verify this possibility, SR threshold measurements should be performed in children with ASD.
